# Characterization of the complete chloroplast genome of *Stellaria dichotoma* var. *lanceolata* Bunge, a traditional Chinese medicinal plant

**DOI:** 10.1080/23802359.2020.1841578

**Published:** 2020-12-24

**Authors:** Peng Li, Pei Yu, Jin Xia

**Affiliations:** Department of Pharmacy, School of Medicine, Xi’an International University, Xi’an, China

**Keywords:** *Stellaria dichotoma* var. *lanceolata* Bunge, Caryophyllaceae, chloroplast genome, Illumina sequencing, phylogenetic analysis

## Abstract

*Stellaria dichotoma* var. *lanceolata* Bunge. is a traditional Chinese medicinal plant of Caryophyllaceae, which contains many chemicals, such as *β*-carboline alkaloids, sterols, cyclic peptides, phenolic acids, and flavonoids and so on, which was used as folk medicines for antifebrile to treat fever and to inhibit the cell growth, cyclooxygenase, and also can anti-inflammatory, anti-allergic, vasodilator, and other effects. Illumina paired-end reads data were used to assemble the complete chloroplast (cp) genome. 16,898,032 raw paired-end reads and the length distribution in 150,602 bp, including a large single-copy (LSC) region of 81,844 bp, a small single-copy (SSC) region of 17,022 bp and a pair of inverted repeats (IRs) of 25,868 bp. Besides, nine protein-coding genes (PCGs) genes and six tRNA genes possess a single intron, while clpP and ycf3 have a couple of introns. Based on the concatenated coding sequences of cp PCGs, the phylogenetic analysis showed that *S. dichotoma* and *Pseudostellaria palibiniana* (MK309611) are closely related to each other within the family Caryophyllaceae.

*S. dichotoma* is a traditional Chinese medicinal plant of Caryophyllaceae, which contains many chemicals, such as *β*-carboline alkaloids, sterols, cyclic peptides, phenolic acids, flavonoids, and other chemical components (Ao et al. 2018; Zhang et al. 2019). *S. dichotoma* was used as folk medicine for antifebrile to treat fever in the late stage of febrile diseases (Yasukawa et al. 1981; Morita et al. 1996). Morita et al. also report that *Stellaria dichotoma* has the activities to inhibit the cell growth and cyclooxygenase (Morita et al. 1996). Some studies have found that *Stellaria dichotoma* also has anti-inflammatory, anti-allergic, vasodilator, and other effects (Wang and You 2012).

The complete chloroplast (cp) genome consists of a pair of inverted repeats (IRs), separated by a large single-copy (LSC) region and a small single-copy (SSC) region, these four parts constitute a conserved structure of the complete cp genome (Wolfe et al. 1992; Lee et al. 2007). This report will be very important for studying the phylogenetic relationships of *Stellaria dichotoma* and Caryophyllaceae.

The fresh leaves of *Stellaria dichotoma* were collected in the Ningxia Forestry Institute (38°28′N, 106°16′E; Ningxia, NW China), then deposited at Pharmaceutical Laboratory in Xi’an International University, the voucher specimen is SD193777. The genomic DNA was extracted with the modified CTAB method (Doyle and Doyle 1987). We constructed a shotgun library with Illumina HiSeq X Ten Sequencing System (Illumina, San Diego, CA) following the manufacturer’s specification. The program MITObim v1.8 (https://github.com/chrishah/MITObim) was used to assemble cp genome (Hahn et al. 2013) and *Pseudostellaria palibiniana* (MK309611) as the initial reference. The map of the complete cp genome was generated through the web-based tool OGDRaw v1.2 (http://ogdraw.mpimp-golm.mpg.de/) (Lohse et al. 2013) and the complete cp genome sequence has been submitted to GenBank (accession number MN718731).

The complete cp genome is a circular double stranded DNA molecule, with a typical quadripartite structure. We assembled a 150,602 bp (GC content accounts for 36.5%) circular cp genome from 16,898,032 raw paired-end reads.

The sequencing result encodes 111 complete genes (the number of 111 did not include the repeat genes), containing 77 protein-coding genes (PCGs), 30 transfer RNA (tRNA) genes, and four ribosomal RNA (rRNA) genes. Six tRNA genes (*trnA-UGC*, *trnG-UCC*, *trnI-GAU*, *trnK-UUU*, *trnL-UAA*, and *trnV-UAC*) harbor a single intron. Nine PCG genes (*atpF*, *ndhA*, *ndhB, petB*, *petD*, *rpl16*, *rpoC1*, *rps12*, and *rps16*) possess a single intron, *clpP* and *ycf3* harbor two introns. In addition, 66 PCG genes have no intron.

Based on the concatenated 10 cp PCGs from 46 published species of Caryophyllaceae, we constructed a Bayesian inference (BI) phylogenetic tree (Figure 1) using MrBayes v3.1.1 (Milne et al. 2009) program integrated with TOPALi V2.5 software (Ronquist and Huelsenbeck 2003) to further study the phylogenetic position of *S dichotoma*. From the BI phylogenetic tree analysis, we find that *S. dichotoma* and *Pseudostellaria okamotoi* (MH879018) are closely related to each other within the family Caryophyllaceae (Figure 1).

**Figure 1.**
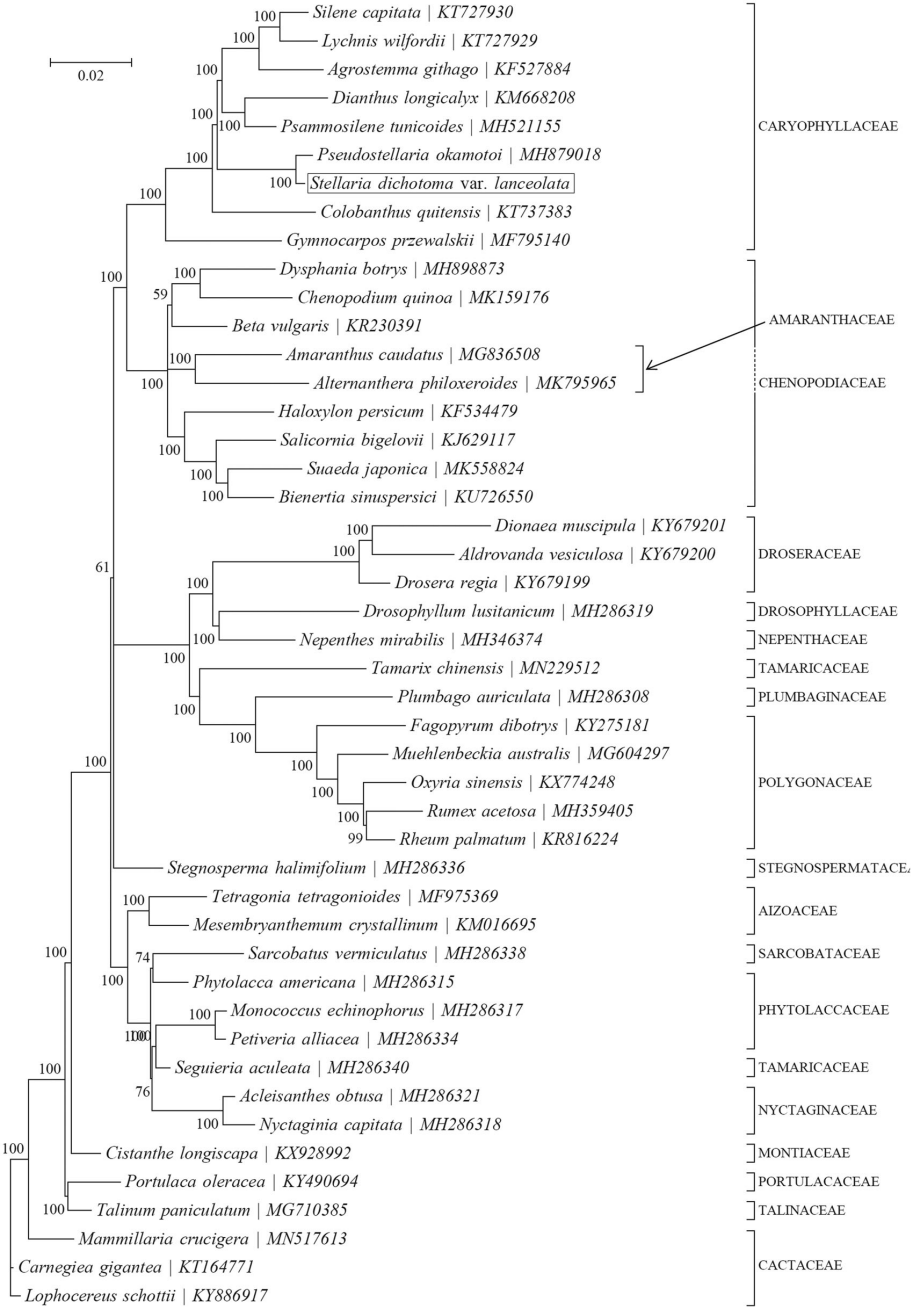
Phylogenetic position of *Stellaria dichotoma* based on a comparison with the complete mitochondrial genome sequences of 45 other Caryophyllaceae species. The analysis was performed using MrBayes v3.1.1 program integrated with TOPALi V2.5 software. The accession number for each species is indicated after the scientific name.

## Data Availability

We confirm that the data supporting the findings of this study are available within the article and its supplementary materials. The data that support the findings of this study are openly available in GenBank of NCBI at https://www.ncbi.nlm.nih.gov, reference number MN718731.
